# Improving clinical suspicion of acute mesenteric ischemia among patients with acute abdomen: a cross-sectional study from an intestinal stroke center

**DOI:** 10.1186/s13017-023-00505-8

**Published:** 2023-06-07

**Authors:** Alexandre Nuzzo, Katell Peoc’h, Prabakar Vaittinada Ayar, Alexy Tran-Dinh, Emmanuel Weiss, Yves Panis, Maxime Ronot, Lorenzo Garzelli, Philippine Eloy, Iannis Ben Abdallah, Yves Castier, Olivier Corcos

**Affiliations:** 1 Université Paris Cité, INSERM UMR 1148, 75018 Paris, France; 2 Université Paris Cité, INSERM UMR 1149, 75018 Paris, France; 3 Department of Gastroenterology, IBD and Intestinal Failure, Intestinal Stroke Center, AP-HP. Nord, Beaujon Hospital, 92110 Clichy, France; 4 Department of Clinical Biochemistry, AP-HP. Nord, Beaujon Hospital, 92110 Clichy, France; 5 Emergency Department, AP-HP. Nord, Beaujon Hospital, 92110 Clichy, France; 6 Intensive Care Unit, AP-HP. Nord, Bichat Hospital, 75018 Paris, France; 7 Intensive Care Unit, AP-HP. Nord, Beaujon Hospital, 92110 Clichy, France; 8 Department of Colorectal Surgery, AP-HP. Nord, Beaujon Hospital, 92110 Clichy, France; 9 Department of Radiology, AP-HP. Nord, Beaujon Hospital, 92110 Clichy, France; 10 Department of Epidemiology, Biostatistics and Clinical Research, APHP. Nord, Bichat Hospital, 75018 Paris, France; 11 Université Paris Cité, INSERM CIC-EC 1425, 75018 Paris, France; 12 Department of Vascular Surgery, AP-HP. Nord, Bichat Hospital, 75018 Paris, France; 13 Structure d’Urgences Vasculaires Intestinales (SURVI), Hôpital Beaujon, 100 bd du général Leclerc, 92110 Clichy, France

**Keywords:** Intestinal ischemia, Colon ischemia, Ischemic colitis, Peritonitis

## Abstract

**Background:**

Early diagnosis of acute mesenteric ischemia (AMI) is essential for a favorable outcome. Selection of patients requiring a dedicated multiphasic computed tomography (CT) scan remains a clinical challenge.

**Methods:**

In this cross-sectional diagnostic study conducted from 2016 to 2018, we compared the presentation of AMI patients admitted to an intestinal stroke center to patients with acute abdominal pain of another origin admitted to the emergency room (controls).

**Results:**

We included 137 patients—52 with AMI and 85 controls. Patients with AMI [median age: 65 years (interquartile range 55–74)] had arterial and venous AMI in 65% and 35% of cases, respectively. Relative to controls, AMI patients were significantly older, more likely to have risk factors or a history of cardiovascular disease, and more likely to present with sudden-onset and morphine-requiring abdominal pain, hematochezia, guarding, organ dysfunction, higher white blood cell and neutrophil counts, and higher plasma C-reactive protein (CRP) and procalcitonin concentrations. On multivariate analysis, two independent factors were associated with the diagnosis of AMI: the sudden-onset (OR = 20, 95%CI 7–60, *p* < 0.001) and the morphine-requiring nature of the acute abdominal pain (OR = 6, 95%CI 2–16, *p* = 0.002). Sudden-onset and/or morphine-requiring abdominal pain was present in 88% of AMI patients versus 28% in controls (*p* < 0.001). The area under the receiver operating characteristic curve for the diagnosis of AMI was 0.84 (95%CI 0.77–0.91), depending on the number of factors.

**Conclusions:**

Sudden onset and the need for morphine are suggestive of AMI in patients with acute abdominal pain and should prompt multiphasic CT scan including arterial and venous phase images for confirmation.

**Supplementary Information:**

The online version contains supplementary material available at 10.1186/s13017-023-00505-8.

## Introduction

Acute mesenteric ischemia (AMI) is a life-threatening cause of acute abdominal pain [1]. AMI accounts for approximately 1% of acute abdominal pain and its incidence was shown to be 8.6/100,000 person-years in a large population-based study [2, 3]. These figures are still considered to be an under-estimation due to diagnostic difficulties. Caused by inadequate blood flow through the mesenteric vessels (either arterial or venous), AMI leads to intestinal necrosis and is associated with 60–80% mortality [1, 2]. Mortality and intestinal resection rates have remained unchanged for decades, despite the progress made in radiology, endovascular procedures, vascular surgery, and intensive care medicine. However, recent implementation of specialized referral centers has suggested improved outcomes for AMI patients when diagnosis and standardized multidisciplinary care are provided at an early stage [4–8]. Indeed, early AMI is a fully reversible condition, as opposed to late AMI, with irreversible transmural necrosis [1, 4, 9].

Timely diagnosis of AMI is critical for ensuring that immediate and appropriate care is provided and thus avoiding death or lifelong complications or impairment, such as short bowel syndrome [10]. However, AMI patients generally present nonspecific acute abdominal pain and biological abnormalities, which renders clinical suspicion and identification challenging, and can often lead to a missed or delayed diagnosis and care [11]. Moreover, no biomarker is currently validated or available [12, 13]. In addition, when clinical suspicion of AMI is not evoked, it may be underdiagnosed on computed tomography (CT) scans of the abdomen as a result of either an inappropriate IV contrast protocol and/or an analysis that does not focus on the mesenteric vessels [14, 15].

This cross-sectional study aimed to compare the clinical and biological presentation of AMI patients to those of other causes of acute abdominal pain to identify diagnostic factors that could help lead to earlier suspicion of the disease.

## Methods

### Study design and setting

Following the results of a pilot study showing an improvement in survival and lower resection rates [4], we created an intestinal stroke center (ISC) that provides 24/7 standardized multimodal and multidisciplinary care to AMI patients referred from the Paris region. Since the creation of this center, we prospectively enrolled AMI patients from the ISC department and control patients who underwent a contrast-enhanced CT-scan for acute abdominal pain from the emergency room (ER) as part of the SURVIBIO cross-sectional diagnostic study. This study was performed following the ethical standards of our institution's Committee on Human Experimentation (Institutional Review Board N°00006477, approval 15-062) and reported according to the Strengthening the Reporting of Observational Studies Epidemiology (STROBE) guidelines [16]. Informed consent was obtained from all patients.

### Patients and controls

**Fig. 1:**
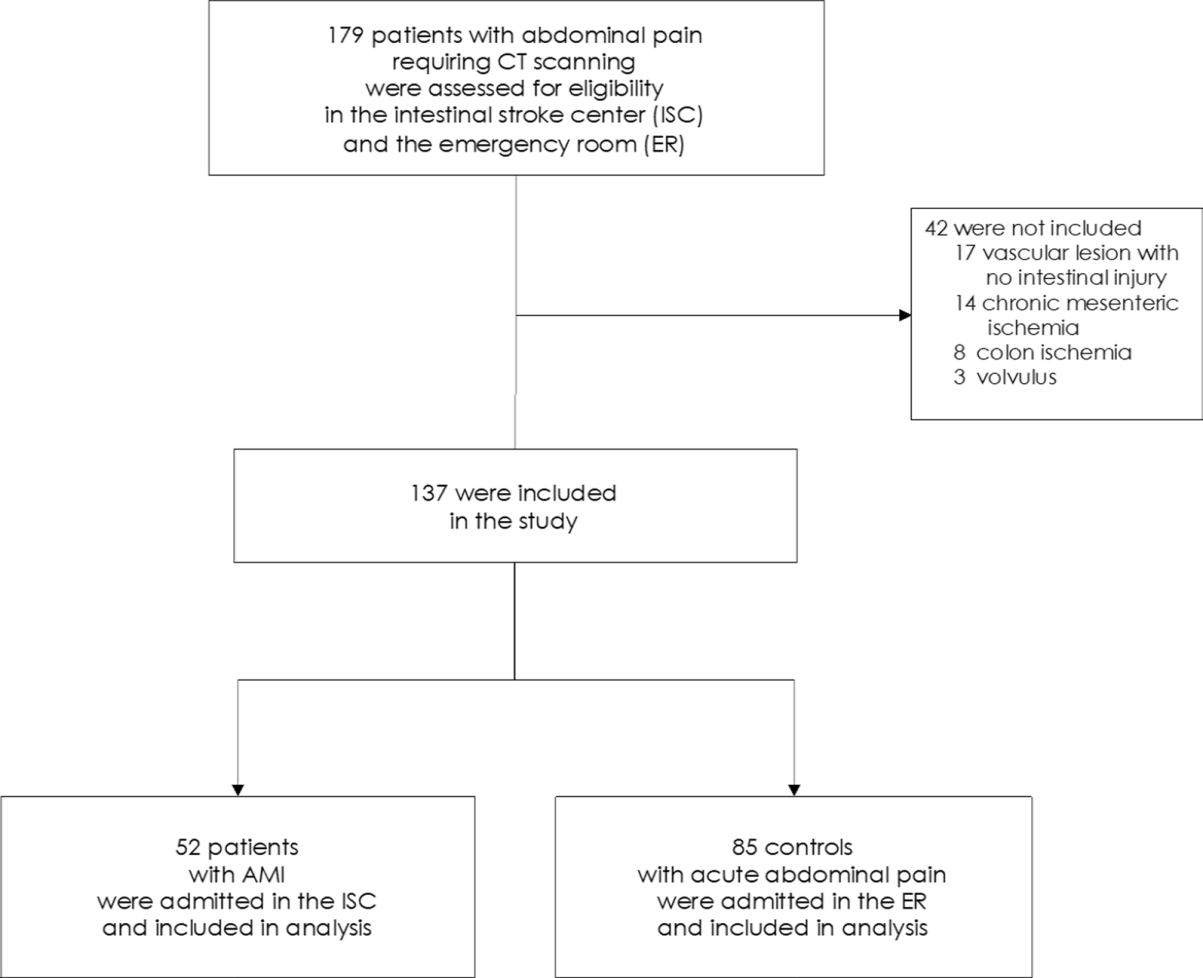
Flowchart of AMI patients and controls: screening and selection

From January 4, 2016, to March 5, 2018, prospective patients who presented with acute abdominal pain requiring a contrast-enhanced multiphasic CT scan to the ER department or referred to the ISC department were evaluated for inclusion in the SURVIBIO diagnostic study. Patients with AMI were admitted to the ISC, whereas those for whom the diagnosis of AMI was ruled out (controls) were admitted to the ER (see patient flowchart, Fig. 1). As previously published [12], the SURVIBIO diagnostic study was originally designed to assess diagnostic biomarkers of AMI. Patients presenting with a diagnosis of left-sided colon ischemia without small bowel injury, chronic mesenteric ischemia without acute injury, vascular lesions with no small bowel injury, or strangulated bowel obstruction were not included so as not to introduce heterogeneity to either the AMI or control groups (see patient flowchart, Fig. 1).

AMI was defined by the association of (1) acute clinical, biological, and contrast-enhanced CT features of bowel injury, (2) vascular insufficiency (occlusive or non-occlusive) of the celiac trunk and/or the superior mesentery artery and/or superior mesenteric vein, and (3) the absence of an alternative diagnosis [5]. The diagnosis of AMI was confirmed or ruled out by the CT scan and alternative final diagnoses were based on clinical, laboratory, and CT findings. Finally, all included patients underwent a multiphasic CT scan including arterial and venous phase images as previously described [17], and a routine biological work-up. Patient clinical records, CT scans, and pathological specimens were reviewed in a monthly multidisciplinary meeting that included gastroenterologists, radiologists, digestive and vascular surgeons, and intensivists, all experts in digestive vascular diseases to avoid misdiagnosis. All CT-scans were reviewed by two senior radiologists specialized in both AMI and digestive diseases (LG and MR).

All AMI patients were managed following a standardized multimodal and multidisciplinary approach in our ISC, as previously described [4]. Briefly, the patients were systematically administered oral antibiotics and antithrombotics [4, 5], and emergency endovascular revascularization of arterial AMI was performed whenever technically feasible. Alternatively, open surgical revascularization was performed. In addition, bowel viability was evaluated following published risk factors for irreversible transmural intestinal necrosis (occurrence of organ failure, elevated serum lactate concentrations, small bowel dilatation, or perforation on CT) [18]. Irreversible transmural intestinal necrosis was confirmed upon pathological assessment.

### Data collection and processing

Routine baseline clinical and biological characteristics were prospectively collected upon admission for all patients: age, gender, history of cardiovascular disease, atherosclerosis risk factors (i.e., tobacco consumption, high blood pressure, diabetes mellitus, or elevated cholesterol or triglycerides), history of venous thromboembolism, history of chronic kidney disease, cirrhosis, ischemic colitis, or abdominal surgery. In addition, the following data concerning AMI was collected: clinical signs at presentation (characteristics of acute abdominal pain, including sudden onset or a requirement for morphine (or other strong opioids), gastrointestinal bleeding, diarrhea, vomiting, constipation, abdominal guarding, sequential organ failure assessment (SOFA) score, and laboratory test values at presentation (white blood cell [WBC], neutrophil, lymphocyte, and platelet counts, the neutrophil-to-lymphocyte ratio [NLR], and hemoglobin, and plasma C-reactive protein [CRP], procalcitonin, l-lactate, blood urea nitrogen, creatinine, aspartate aminotransferase, and bilirubin levels). Morphine-requiring abdominal pain was defined as a pain unrelieved by weak opioids (such as tramadol or codeine) and relieved by > 2 mg intravenous morphine (or equivalent). Pain relief was defined as ≤ 30/100 mm on the visual analogue pain scale. The sudden-onset was defined by an abdominal pain that started and peaked within an hour or less. The origin of AMI (arterial–thrombotic or embolic–venous, or non-occlusive) was specified based on the patient records, CT scan, and pathological review.

### Statistical analysis

Categorical variables, expressed as counts (percentages) and frequency distributions, were compared between groups using Chi square or Fisher exact tests, as appropriate. Continuous variables are expressed as medians [interquartile ranges (IQR)] and were compared between groups using Student t or Mann–Whitney U tests, as appropriate. Associations between the clinical and biological presentation and the diagnosis of AMI were assessed through multivariate logistic regression models. The main model included the following covariates: sudden-onset and morphine-requiring abdominal pain, abdominal guarding, SOFA score > 2, and WBC. A series of sensitivity analyses were performed to assess the robustness of the findings (Additional file 1). Models with further adjustments for age, history of cardiovascular disease, atherosclerosis risk factors, hematochezia, CRP and procalcitonin were also tested. Multicollinearity between selected variables was assessed using the variance inflation factor (VIF). Variables were considered to be suspicious for collinearity when the VIF was > 5 [19]. Neutrophil counts were excluded from the multivariate model because of collinearity with white blood cell counts. All other covariates were included in the model and no variable selection was performed. Results of the multivariate analysis are shown as odds ratios (ORs) (95% confidence interval) and were used to compute a score according to the number of independent factors. The accuracy of the resulting score was further evaluated using the area under the receiver operating characteristic (AUROC) curve, sensitivity, specificity, and positive/negative likelihood ratios. All tests were two-sided. A *p*-value < 0.05 was considered significant. No imputation of missing data was performed. All analyses were performed using the Statistical Package for the Social Sciences (SPSS) for Mac OSX software (version 23.0, Chicago, IL, USA) and the pROC package in R software, version 3.6.2 (R Foundation for Statistical Computing) [20].

## Results

### Characteristics of the SURVIBIO population

Between January 4, 2016, and March 5, 2018, 179 patients with acute abdominal pain who underwent a contrast-enhanced CT-scan were assessed for eligibility (Fig. 1). We enrolled 137 patients, including 52 admitted to our ISC for AMI and 85 admitted to the ER for acute abdominal pain of another origin (see flowchart, Fig. 1). The baseline characteristics of both populations are summarized in Table 1. Patients with AMI [median age: 65 years (IQR 55–74), 37% women] included arterial and venous causes in 65% and 35% of cases, respectively. None of the included patients had non-occlusive AMI. AMI occurred in seven patients with a prior history of chronic mesenteric ischemia. The control group included patients with the following diagnoses: infectious disease (*n* = 20; 10 cases of diverticulitis, five of appendicitis, five others), abdominal inflammatory diseases (*n* = 15; eight intra-abdominal neoplasms, seven inflammatory bowel disease flares), small bowel mechanical obstruction (*n* = 13), functional gastrointestinal disease (*n* = 13), pancreatic or biliary syndromes (*n* = 12), urological or genital causes (*n* = 11), and one patient with abdominal pain related to invasive meningococcemia. After admission to the ISC, AMI patients received antiplatelet therapy (*n* = 34, 100% arterial AMI), anticoagulants (*n* = 51, 98%), oral antibiotics (*n* = 51, 98%), and intravenous antibiotics (*n* = 21, 40%). Emergency revascularization was performed on 30 patients (88% of arterial AMI patients) after a median of 14 h. During the follow-up period, 16 of the AMI patients (31%) required a laparotomy, confirming transmural intestinal necrosis in 14 patients (27%), and 38 patients (73%) recovered with no need for intestinal resection. Mortality at 12 months was 13%, 18%, and 5% in the overall cohort and the arterial AMI and venous AMI groups, respectively.

**Table 1:** Admission characteristics of patients with acute mesenteric ischemia (AMI) or abdominal pain of other cause (controls)

	AMI patients *N* = 52 (%)	Controls *N* = 85 (%)	*p*-value
Age, years^a^	65 (55–74)	48 (35–70)	< 0.001
Female	19 (37)	34 (40)	0.69
**Atherosclerosis risk factors (at least one)**	42 (81)	38 (45)	< 0.001
Tobacco use	24 (46)	17 (20)	
Arterial hypertension	29 (56)	23 (27)	
Dyslipidemia	20 (39)	12 (14)	
Diabetes mellitus	12 (23)	5 (6)	
**Cardiovascular history (at least one)**	34 (65)	20 (24)	< 0.001
Myocardial ischemia	10 (19)	5 (6)	
Stroke	6 (12)	5 (6)	
Limb ischemia	9 (17)	2 (2)	
Atrial fibrillation	11 (21)	4 (5)	
Heart surgery	3 (6)	1 (1)	
Vascular surgery	13 (25)	1 (1)	
Deep vein thrombosis	4 (8)	4 (5)	
Pulmonary embolism	6 (12)	4 (5)	
**Other comorbidities**			
Chronic kidney disease	1 (2)	2 (2)	1.00
Colon ischemia	0 (0)	1 (1)	1.00
Cirrhosis	4 (8)	4 (5)	0.48
Abdominal surgery	29 (56)	40 (47)	0.32
History of digestive neoplasm	7 (13)	19 (22)	0.20
**Clinical features**			
Temperature^a^	37.0 (36.3–37.1)	36.8 (36.5–37.5)	0.52
Mean arterial pressure^a^	99.8 (89.1–110.1)	96.0 (84.3–107.2)	0.35
Heart rate^a^	88 (76–104)	86 (71–104)	0.35
Sudden-onset abdominal pain	31 (71)	9 (11)	< 0.001
Morphine-requiring abdominal pain	33 (64)	19 (22)	< 0.001
Ileus	8 (15)	14 (17)	0.87
Vomiting	21 (40)	41 (48)	0.37
Diarrhea	12 (23)	13 (15)	0.25
Hematochezia	8 (15)	3 (4)	0.02
Guarding	17 (33)	16 (19)	0.07
Total SOFA score > 2	15 (30)	8 (10)	0.004

*Abbreviations: AMI*, acute mesenteric ischemia, *SOFA*, sequential organ failure assessment score

^a^Median (interquartile range)

### Factors associated with the diagnosis of AMI

**Fig. 2:**
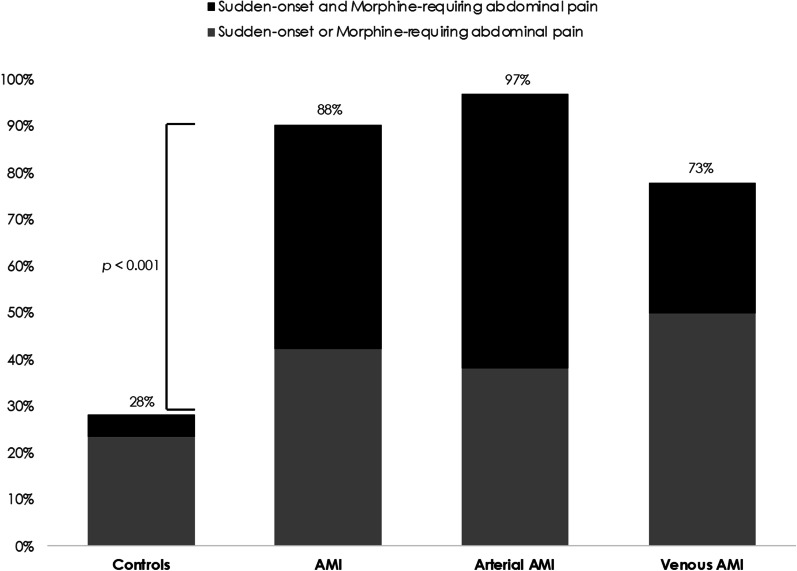
Prevalence of sudden-onset and/or morphine-requiring abdominal pain signs in AMI patients and controls. Sudden-onset and/or morphine-requiring presenting abdominal pain was present in 28% of controls versus 88%, 97%, and 73% of AMI, arterial and venous AMI patients, respectively (*p* < 0.001)

Patients with AMI were significantly older and were more likely to have risk factors or a history of cardiovascular disease than those with non-ischemic abdominal pain (Table 1). At admission, AMI patients were also more likely to present with sudden-onset and/or morphine-requiring abdominal pain, hematochezia, guarding, organ dysfunction, higher white blood cell and neutrophil counts, and CRP and plasma procalcitonin concentrations. Other admission clinical and laboratory characteristics, including l-lactate levels, did not significantly differ (Tables 1, 2). Notably, AMI patients had normal plasma lactate concentrations (< 2 mmol/L) upon admission in 69% of cases (*n* = 36). The AUROC curve for the diagnosis of AMI of white blood cell and neutrophil counts and plasma CRP and procalcitonin were 0.61 (95%CI 0.52–0.71, *p* = 0.02), 0.61 (95%CI 0.51–0.71, *p* = 0.03), 0.73 (95%CI 0.64–0.82, *p* < 0.001), and 0.74 (95%CI 0.64–0.83, *p* < 0.001), respectively (Additional file 1: Fig. S1). On multivariate analysis (Table 3), two independent factors were associated with the diagnosis of AMI: the sudden onset (OR = 20.2, 95%CI 6.9–59.6, *p* < 0.001) and the morphine-requiring nature of the acute abdominal pain (OR = 5.5, 95%CI 1.9–15.9, *p* = 0.002). Overall, results remained similar in all sensitivity analyses (Additional file 1: Tables). Sudden-onset and/or morphine-requiring presenting abdominal pain was present in 88% of AMI (97% of arterial AMI and 73% of venous AMI) versus 28% in controls (*p* < 0.001, Fig. 2). The overall AUROC curve for the diagnosis of AMI was 0.84 (95%CI 0.77–0.91), depending on the number of diagnostic factors (Table 4; Additional file 1: Fig. S2). The associated sensitivity, specificity, and positive and negative likelihood ratios are shown in Table 4.

**Table 2:** Admission biological characteristics of patients with acute mesenteric ischemia (AMI) or abdominal pain of other cause (controls)

	AMI patients *N* = 52	Controls *N* = 85	*p*-value
White blood cell count, G/L	12 (9–18)	11 (8–14)	0.02
Neutrophils, G/L	10 (7–14)	8 (5–11)	0.03
Lymphocytes, G/L	1.2 (0.7–1.6)	1.3 (0.9–1.9)	0.15
Neutrophil–lymphocyte ratio (NLR)	8 (5–17)	6 (3–12)	0.11
Platelet count, G/L	266 (171–363)	271 (− 319)	0.46
Hemoglobin, g/dL	12.5 (11.6–15.1)	13.6 (12.3–14.9)	0.21
C-reactive protein, mg/L	107 (30–205)	21 (5–98)	< 0.001
Procalcitonin, ng/mL	0.4 (0.1–1.2)	0.1 (0.0–0.2)	< 0.001
Lactate, mmol/L	1.5 (1.0–2.3)	1.4 (1.0–2.2)	0.68
Urea, mmol/L	6 (4–9)	5 (4–7)	0.10
Creatinine, µmol/L	72 (63–100)	70 (63–89)	0.76
ASAT, UI/L	25 (19–38)	27 (22–40)	0.23
Bilirubin, µmol/L	12 (8–19)	12 (9–18)	0.74

Values are medians (interquartile range)

**Table 3:** Multivariate analysis of factors associated with the diagnosis of acute mesenteric ischemia

	Logistic regression model		
	*p*-value^a^	OR	(95% CI)
Sudden onset of abdominal pain	< 0.001	20.2	(6.9–59.6)
Morphine-requiring abdominal pain	0.002	5.5	(1.9–15.9)
Guarding	0.63	–	–
Organ dysfunction (total SOFA score > 2)	0.24	–	–
White blood cell count, G/L	0.11	–	–

The multivariate model included all 5 variables and 129 complete cases. All other covariates were included in the model and no variable selection was performed

*OR*, odds ratio, *CI*, confidence interval, *AMI*, acute mesenteric ischemia

^a^Wald test

**Table 4:** Diagnostic accuracy of sudden-onset and/or morphine-requiring acute abdominal pain signs in acute mesenteric ischemia

Population	AUROC (95%CI)^a^	Abdominal pain criteria	Sensitivity (95%CI)^a^	Specificity (95%CI)^a^	Positive likelihood ratio (95%CI)^a^	Negative likelihood ratio (95%CI)^a^
Overall	0.84 (0.77–0.91)	Sudden-onset	0.73 (0.61–0.85)	0.89 (0.83–0.96)	6.90 (3.64–13.08)	0.30 (0.19–0.47)
		Morphine-requiring	0.63 (0.50–0.77)	0.78 (0.69–0.87)	2.84 (1.82–4.44)	0.47 (0.32–0.69)
		1 factor	0.88 (0.80–0.97)	0.72 (0.62–0.81)	3.13 (2.20–4.46)	0.16 (0.07–0.35)
		2 factors	0.48 (0.34–0.62)	0.95 (0.91–1.00)	10.22 (3.77–27.70)	0.54 (0.42–0.71)
Arterial AMI	0.89 (0.82–0.96)	Sudden-onset	0.88 (0.77–0.99)	0.89 (0.83–0.96)	8.33 (4.44–15.64)	0.13 (0.05–0.33)
		Morphine-requiring	0.68 (0.52–0.83)	0.78 (0.69–0.87)	3.03 (1.91–4.79)	0.42 (0.25–0.69
		1 factor	0.97 (0.91–1.00)	0.72 (0.62–0.81)	3.44 (2.44–4.85)	0.04 (0.006–0.28)
		2 factors	0.59 (0.42–0.75)	0.95 (0.91–1.00)	12.50 (4.61–33.88)	0.43 (0.29–0.65)
Venous AMI	0.75 (0.62–0.88)	Sudden-onset	0.42 (0.20–0.64)	0.89 (0.83–0.96)	4.00 (1.77–8.96)	0.65 (0.44–0.96)
		Morphine-requiring	0.58 (0.36–0.80)	0.78 (0.69–0.87)	2.59 (1.49–4.50)	0.54 (0.32–0.93)
		1 factor	0.74 (0.54–0.93)	0.72 (0.62–0.81)	2.61 (1.69–4.02)	0.37 (0.17–0.79)
		2 factors	0.26 (0.07–0.46)	0.95 (0.91–1.00)	5.59 (1.66–18.89)	0.77 (0.59–1.00)

^a^95%CI - 95% confidence interval

## Discussion

In this cross-sectional study of 137 patients with acute abdominal pain, including a large proportion with AMI in its early stages, we identified two independent factors—the sudden-onset and the morphine-requiring nature of the abdominal pain—associated with the diagnosis of AMI. These two clinical signs are readily observable in clinical practice upon admission and showed high AUROC values and high positive and negative likelihood ratios for the diagnosis of AMI. Considering an estimated prevalence of AMI of 1% among acute abdominal pain patients [2, 3], the probability of AMI would be approximately 0.01% or 10% for a patient with no or two factors, respectively [21]. Thus, in the current absence of an accurate diagnostic tool or biomarker [12, 13], we believe these factors may help emergency physicians, gastroenterologists, radiologists, intensivists, and digestive and vascular surgeons raise the suspicion of AMI earlier among patients with acute abdominal pain and lead to an urgent investigation by a dedicated abdominal multiphasic CT scan clearly motivated by the suspicion of ischemia.

AMI is often overlooked by physicians in its early stages due to the paucity of clinical and biological abnormalities and often diagnosed late, when the treatment outcome is inevitably poor [1, 2, 7]. As a result, previous studies mostly enrolled severe AMI patients at a late transmural infarction surgical stage [22, 23]. These studies also frequently included and merged patients with AMI and those with heterogeneous conditions such as strangulated bowel obstructions or left-side colon ischemia with no small bowel injury. We believe the results of such study designs may be misleading, overestimating the diagnostic accuracy of late clinical signs or biomarkers, such as l-lactate or procalcitonin levels or the NLR for the diagnosis of AMI [13, 24–26]. Therefore, their results cannot be used to diagnose AMI in its early stages, when improved outcomes and survival are possible. Instead, our study included a homogeneous population of 52 well-defined patients with confirmed arterial and venous AMI, treated with a standardized care protocol in an intestinal stroke center, enrolled on admission, at the time of diagnosis, and at an early stage in 73% of cases.

As expected, we observed significantly higher rates of cardiovascular history and risk factors for AMI patients than controls. However, these epidemiological factors were no longer associated with the diagnosis of AMI in multivariate analysis (sensitivity analyses, see Additional file 1). Although AMI patients were two times more likely to have cardiovascular risk factors and three times more likely to have prior cardiovascular history, they were also significantly older than controls. Most importantly, one-third of AMI patients had no history of cardiovascular disease whatsoever. These observations are consistent with those reported by Adaba et al*.* in a U.K. retrospective cohort [27]. As previously described [28–31], acute abdominal pain of AMI patients was commonly out of proportion to physical examination, as more than 65% of patients reported a sudden onset and required morphine treatment. Nevertheless, AMI patients presented without severe signs in most cases, with no reporting of abdominal guarding in 67% of cases and no organ failure in 70% of cases. Similarly, in the study of Kougias et al., peritonitis and shock were reported on admission in only 36% and 6% of cases, respectively [31]. Overall, these results are another reminder that AMI patients commonly present at an early and potentially reversible stage. Elevated plasma lactate levels and organ failure are late findings associated with intestinal necrosis [18]. Of note, plasma lactate concentrations were initially within the normal range in our cohort of AMI patients, thus confirming their diagnostic inutility in the early stages of AMI. Indeed, in a retrospective cohort study of survivors from mesenteric infarction, a delayed diagnosis was more frequent when initial plasma lactate concentrations were < 2 mmol/L, suggesting that physicians might be misguided by such unremarkable lactate levels [15].

Despite constant improvements in diagnostic, interventional, and surgical techniques, AMI remains a life-threatening emergency with high mortality rates. Misdiagnosis or a delayed diagnosis are the most important predictors of patient outcomes. Indeed, no specific clinical or biological signs have proven to be sufficiently sensitive or specific to suggest the diagnosis in the emergency setting [1, 9, 13, 26]. As a result, the early recognition of AMI is still a major clinical challenge and relies on a high index of clinical and radiological suspicion before confirmation on a multiphasic CT scan [32]. Although CT-scan is widely available in the emergency setting and reported to have excellent performance for AMI diagnosis, timely clinical suspicion and the selection of patients requiring a CT scan is still challenging [32]. Furthermore, lower sensitivity of CT scans has been observed in real-life clinical settings when the CT-scan protocol does not include both arterial and venous phases [14, 33, 34]. In underdiagnosed or doubtful cases, explorative laparoscopy can then help confirm the diagnosis [35], and signs of irreversible transmural intestinal necrosis (organ failure, elevated serum lactate concentrations, small bowel dilatation, or perforation on CT) should prompt laparotomy [18]. Based on observational studies, the recent guidelines recommend that “severe abdominal pain out of proportion to physical examination findings should be assumed to be AMI until disproven” [28, 35]. As a reminder of the importance of the clinical exam and clinical suspicion in the diagnosis of AMI, our study provides prospective and comparative evidence further supporting this recommendation, and suggests that suspicion of an AMI diagnosis be raised in the presence of any acute abdominal pain when at least one of these two factors is present: (1) a reported sudden onset or (2) the requirement for morphine. As the discriminative weight of epidemiological and biological factors was insufficient, we suggest the diagnosis be evoked irrespective of the patients’ age, prior cardiovascular history, or laboratory values (such as CRP or l-lactate levels) [14]. In the absence of a validated available diagnostic biomarker of AMI, broad and timely clinical suspicion and subsequent confirmation by an appropriate multiphasic CT scan is currently the only way to achieve an earlier diagnosis and better outcomes [1, 28].

Certain limitations of our study merit discussion. First, this study was originally designed to assess biomarkers of AMI. As a result, we did not include patients with strangulated bowel obstruction, left-sided colon ischemia, or mesenteric vessel occlusion without evidence of acute small bowel injury in either the patient or control groups. This may have decreased the generalizability of our findings for the discrimination of AMI from these other excluded conditions, although these conditions may share pathophysiological ischemic processes comparable to those of AMI. However, they are different diseases with different prognoses and their inclusion could have introduced biases in comparing AMI patients and controls. Nonetheless, our work represents one of the largest prospective populations of well-characterized and homogeneous AMI patients, including a large proportion of early forms. This is crucial, as the question of the early diagnosis of AMI could not be addressed by studying AMI in its late-stage. Our findings highlight two clinical factors associated with the diagnosis that may be discriminant. However, one can argue that the sudden onset characteristic of the pain and the morphine requirement may be partially subjective. Finally, as a referring hospital for AMI, the prevalence of AMI was high in this cross-sectional study and, thus, the predictive values could not be calculated.

## Conclusion

In conclusion, this cross-sectional study identified two independent clinical factors associated with a diagnosis of AMI that may help physicians suspect the disease earlier among patients presenting with acute abdominal pain. As a timely diagnosis remains the most critical determinant of patient outcomes, our result suggests questioning AMI in any patients presenting with acute abdominal pain of sudden onset and/or requiring morphine, and prompt multiphasic CT scan angiography including arterial and venous phase images for confirmation. However, further studies are required to confirm the diagnostic value of these factors and improve objective means to achieve an earlier diagnosis.

## Supplementary Information

**Additional file 1**. Tables A1 and A2.

## Data Availability

Research data are not shared.

## Abbreviations

AMI: Acute mesenteric ischemia
CT: Computed tomography
ISC: Intestinal stroke center
SOFA: Sequential organ failure assessment
WBC: White blood cell
NLR: Neutrophil-to-lymphocyte ratio
CRP: Plasma C-reactive protein
IQR: Interquartile ranges
VIF: Variance inflation factor
OR: Odds ratios
AUROC: Area under the receiver operating characteristic curve
