# Supplementary material for: Improving clinical suspicion of acute mesenteric ischemia among patients with acute abdomen: a cross-sectional study from an intestinal stroke center
Source: World J Emerg Surg. 2023 Jun 7;18:37. doi: 10.1186/s13017-023-00505-8 (PMC10246417; doi:10.1186/s13017-023-00505-8)
Supplement: Supplementary file 1 — Additional file 1. Tables A1 and A2. [file 13017_2023_505_MOESM1_ESM.docx]

**Online supplementary materials**

**Improving clinical suspicion of acute mesenteric ischemia among patients with acute abdominal pain: a cross-sectional study from an intestinal stroke center.**

Authors: Alexandre Nuzzo MD PhD (1,3)*, Katell Peoc'h PharmD PhD (2,4), Prabakar Vaittinada Ayar MD (5), Alexy Tran-Dinh MD PhD (1,6), Emmanuel Weiss MD PhD (2,7), Yves Panis MD PhD (8), Maxime Ronot MD PhD (4,9), Lorenzo Garzelli MD (4,9), Philippine Eloy MD (10,11), Iannis Ben Abdallah MD (12), Yves Castier MD PhD (12), and Olivier Corcos MD (1,3)

*[al.nuzzo@gmail.com](mailto:al.nuzzo@gmail.com)

**Figure A. Common laboratory values on admission of acute mesenteric ischemia patients and controls**

Relative to controls, AMI patients had higher white blood cell counts (p = 0.02), neutrophil counts ( p =0.03), and plasma C-reactive protein levels (p < 0.001) and procalcitonin levels (p < 0.001).

AMI: acute mesenteric ischemia, G/L: giga per liter, mg/L: milligram per liter, ng/mL: nanogram per milliliter

**Figure B. Area under the receiver operating characteristic curve of the sudden-onset and/or morphine-requiring acute abdominal pain signs in acute mesenteric ischemia**

**Tables A: Factors associated with the diagnosis of acute mesenteric ischemia – sensitivity analyses**

Table A1: main model, additionally adjusted for hematochezia (categorical: yes/no), C-reactive protein (continuous, mg/L), and procalcitonin (continuous, ng/L)

|  | Logistic regression model | | |
| --- | --- | --- | --- |
|  | *p-value^1^* | OR | (95% CI) |
|  |  |  |  |
| **Sudden onset of abdominal pain** | **< 0.001** | **21.3** | **(5.7 – 79.3)** |
| **Morphine-requiring abdominal pain** | **0.02** | **4.8** | **(1.3 – 17.4)** |
| Hematochezia | 0.72 | - | - |
| Guarding | 0.34 | - | - |
| Organ dysfunction (total SOFA score > 2) | 0.41 | - | - |
| White blood cell count, G/L | 0.47 | - | - |
| C-reactive protein, mg/L | 0.06 | - | - |
| Procalcitonin, ng/L | 0.15 | - | - |
|  |  |  |  |

^1^ Wald test. The multivariate model included all 8 variables and 102 complete cases. All other covariates were included in the model and no variable selection was performed. OR: odds ratio, CI: confidence interval, AMI: acute mesenteric ischemia

Table A2: main model, additionally adjusted for hematochezia (categorical: yes/no), C-reactive protein (continuous, mg/L), and procalcitonin (continuous, ng/L), age (continuous, years), atherosclerosis risk factors (at least one, categorical: yes/no), cardiovascular history (at least one, categorical: yes/no)

|  | Logistic regression model | | |
| --- | --- | --- | --- |
|  | *p-value^1^* | OR | (95% CI) |
|  |  |  |  |
| Age | 0.84 | - | - |
| Atherosclerosis risk factors (at least one) | 0.30 | - | - |
| Cardiovascular history (at least one) | 0.38 | - | - |
| **Sudden onset of abdominal pain** | **< 0.001** | **16.2** | **(4.0 – 65.8)** |
| **Morphine-requiring abdominal pain** | **0.04** | **4.0** | **(1.0 – 15.5)** |
| Hematochezia | 0.74 | - | - |
| Guarding | 0.49 | - | - |
| Organ dysfunction (total SOFA score > 2) | 0.44 | - | - |
| White blood cell count, G/L | 0.54 | - | - |
| C-reactive protein, mg/L | 0.10 | - | - |
| Procalcitonin, ng/L | 0.12 | - | - |
|  |  |  |  |

^1^ Wald test. The multivariate model included all 11 variables and 102 complete cases. All other covariates were included in the model and no variable selection was performed. OR: odds ratio, CI: confidence interval, AMI: acute mesenteric ischemia
